# Effects of Ambient Temperature on the Mechanical Properties of Frictionally Welded Components of Polycarbonate and Acrylonitrile Butadiene Styrene Dissimilar Polymer Rods

**DOI:** 10.3390/polym15173637

**Published:** 2023-09-02

**Authors:** Chil-Chyuan Kuo, Naruboyana Gurumurthy, Song-Hua Huang

**Affiliations:** 1 Department of Mechanical Engineering, Ming Chi University of Technology, No. 84, Gungjuan Road, Taishan District, New Taipei City 24301, Taiwan; 2 Research Center for Intelligent Medical Devices, Ming Chi University of Technology, No. 84, Gungjuan Road, Taishan District, New Taipei City 24301, Taiwan; 3 Department of Mechanical Engineering, Chang Gung University, No. 259, Wenhua 1st Road, Guishan District, Taoyuan City 33302, Taiwan; 4 Center of Reliability Engineering, Ming Chi University of Technology, No. 84, Gungjuan Road, Taishan District, New Taipei City 24301, Taiwan; 5 Department of Mechanical Engineering, Presidency University, Rajankunte, Near Yelhanka, Bangalore 700073, India; 6 Li-Yin Technology Co., Ltd., No. 37, Lane 151, Section 1, Zhongxing Road, Wugu District, New Taipei City 24101, Taiwan

**Keywords:** rotary friction welding, ambient temperature, heat-affected zone, bending strength, impact energy

## Abstract

Rotary friction welding (RFW) has no electric arc and the energy consumption during welding can be reduced as compared with conventional arc welding since it is a solid-phase welding process. The RFW is a sustainable manufacturing process because it provides low environmental pollution and energy consumption. However, few works focus on the reliability of dissimilar polymer rods fabricated via RFW. The reliability of the frictionally welded components is also related to the ambient temperatures. This work aims to investigate the effects of ambient temperature on the mechanical properties of frictionally welded components of polycarbonate (PC) and acrylonitrile butadiene styrene (ABS) dissimilar polymer rods. It was found that the heat-affected zone width increases with increasing rotational speeds due to peak welding temperature. The Shore A surface hardness of ABS/PC weld joint does not change with the increased rotational speeds. The Shore A surface hardness in the weld joint of RFW of the ABS/PC is about Shore A 70. The bending strength was increased by about 53% when the welded parts were placed at 60–70 °C compared with bending strength at room temperature. The remarkable finding is that the bending fracture position of the weldment occurs on the ABS side. It should be pointed out that the bending strength can be determined by the placed ambient temperature according to the proposed prediction equation. The impact energy was decreased by about 33% when the welded parts were placed at 65–70 °C compared with the impact energy at room temperature. The impact energy (y) can be determined by the placed ambient temperature according to the proposed prediction equation. The peak temperature in the weld interface can be predicted by the rotational speed based on the proposed equation.

## 1. Introduction

Rotary friction welding (RFW) [1] is a practical solid-state joining technique that is extensively employed in many industries [2], including automotive [3], aerospace [4], and marine industries [5]. Significantly, RFW has lower energy consumption and environmental impact as compared with arc welding. This technology is commonly used in the aircraft and electrical industries. Thus, it is frequently used to manufacture naval, mining, automotive, shafts, tubes, piston rods, or truck roller bushes. RFW requires very little heat and friction to the components while welding and helps to lessen the grain formation [6]. This technology can join metals to plastics using a machined metal interface [7].

Khalaf et al. [8] found that the heat generated in pins with more edges and triangular shapes was more significant than the pins with a smooth shape, showing the higher heat generation caused by the heat flux on the surface of the high-density polyethylene. Vidakis et al. [9] investigated the travel speed, welding tool pin geometry, and rotational speed of acrylonitrile butadiene styrene (ABS) manufactured via the material extrusion process [10]. The results showed that the welded specimens exhibited increased mechanical strength compared to non-welded three-dimensional printed specimens of the same geometry. Yang et al. [11] analyzed the contact behavior and temperature characterization during welding using the harmonic balance method. The simulation and experimental results revealed that welding time and amplitude are critical interface temperature factors. The interface temperature can be increased significantly by increasing the welding time and amplitude. Maggiore et al. [12] reviewed the structural adhesive joints in hybrid joining processes. The result revealed that utilizing hybrid joining technology is a potential method for reducing manufacturing costs and mass in various industries. Pereira et al. [13] found that increasing the rotational speed/welding speed ratio increased the joint efficiency. It was observed that it is not easy to establish mathematical relationships because the variability of welding conditions. Iftikhar et al. [14] classified the literature on the friction stir spot welding and friction stir welding of thermoplastic polymers and polymer composites based on tooling conditions, joining materials, joint configurations, and medium conditions. Ma et al. [15] found a reduction in the gradient along the thickness. This is because of material flow at the bottom and the increased pinhole heat input. Skowrońska et al. [16] investigated the structural properties of welded joints using high-speed friction welding. The results showed that the surface hardness in the weld joint exceeding HV 340 was obtained. Eliseev et al. [17] found that the grain size of incoherent intermetallic particles and the volume fraction were decreased towards the center of the layer in the transfer layer of aluminum alloy welds. It was Anwar et al. [18] studied the microstructure of the alloy 800H rotary friction welds in post-weld heat-treated. The results showed that post-weld heat treatment successfully met the minimum grain size with improved strength and elongation. Meng et al. [19] reviewed the current progress regarding friction-based welding techniques, containing joining mechanism, welding tool design, technical development, microstructural characteristics, process optimization, and surface modification. Huang et al. [20] proposed a new technique of friction-based filling stacking joining for polymer and metal. The maximum tensile shear strength of 13 MPa was obtained. This method shows that the proposed filling stacking welding could join thermoplastic and metal. Meng et al. [21] proposed friction self-riveting welding to build heterojunctions between metals and polymer matrix composites. The maximum tensile shear strength of 27 MPa was obtained.

Polycarbonate (PC) [22] and ABS [23] plastics are extensively employed in some components of consumer electronics since these are lightweight than metal. PC plastic belongs to engineering thermoplastics because it has excellent heat resistance. Naturally, the ABS plastic has high tensile strength, highly resistant to chemical corrosion, and physical impacts. Thus, ABS plastic is suitable to produce products with excellent mechanical properties. However, rare studies focus on the reliability of PC/ABS dissimilar polymer rods fabricated via RFW. Based on years of practical experience, the validity and reliability [24,25] of the frictionally welded components are related to the ambient temperatures significantly [26]. Therefore, the investigation of mechanical properties [27] of frictionally welded components at different environmental temperatures is an important research topic. The main objective is to investigate the effects of ambient temperature on the mechanical properties of frictionally welded components of PC/ABS dissimilar polymer rods. The three-point bending test, impact tests, and shore A surface hardness tests were performed to investigate the welding quality. After bending and impact tests, fracture surfaces were investigated using field emission scanning electron microscopy (FE-SEM) and optical microscope (OM). Finally, a database of RFW of PC and ABS dissimilar polymer rods was established.

## 2. Experimental Details

**Figure 1:**
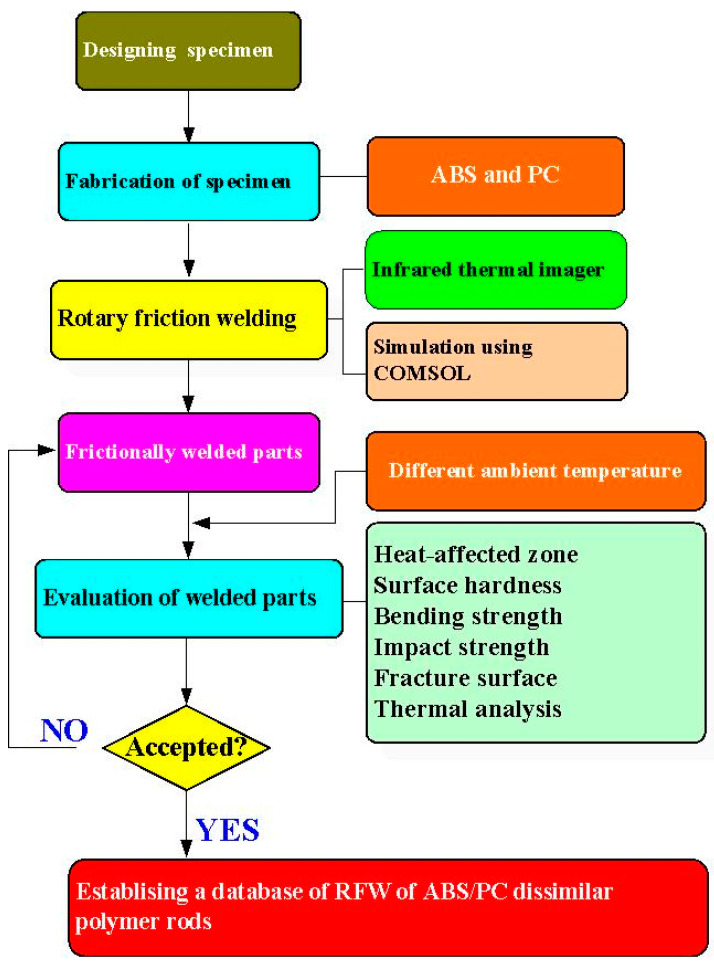
Flowchart of the research process in this study.

**Figure 2:**
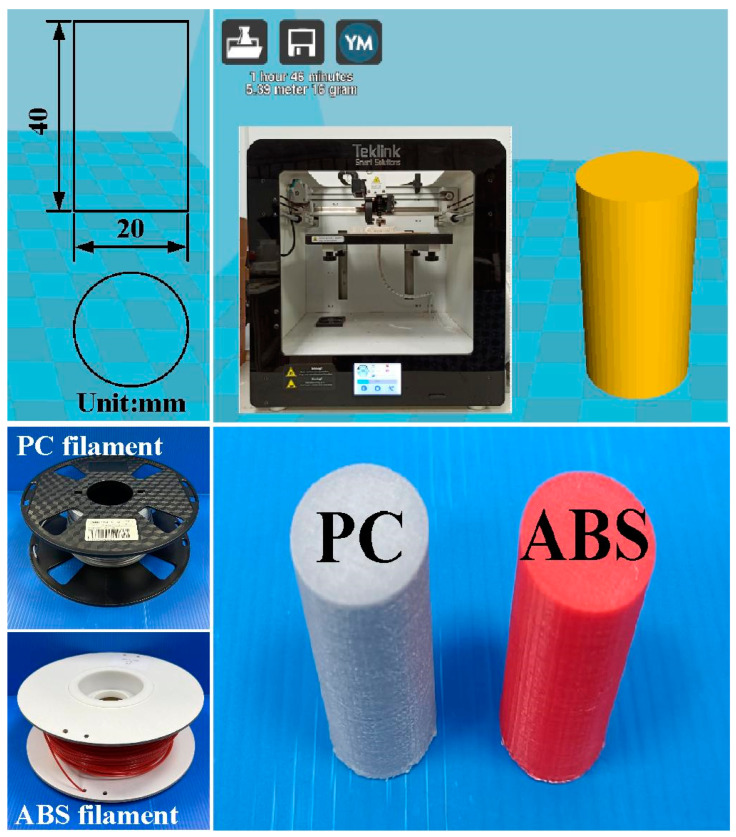
Welding specimens for RFW of ABS and PC dissimilar polymer rods.

**Figure 3:**
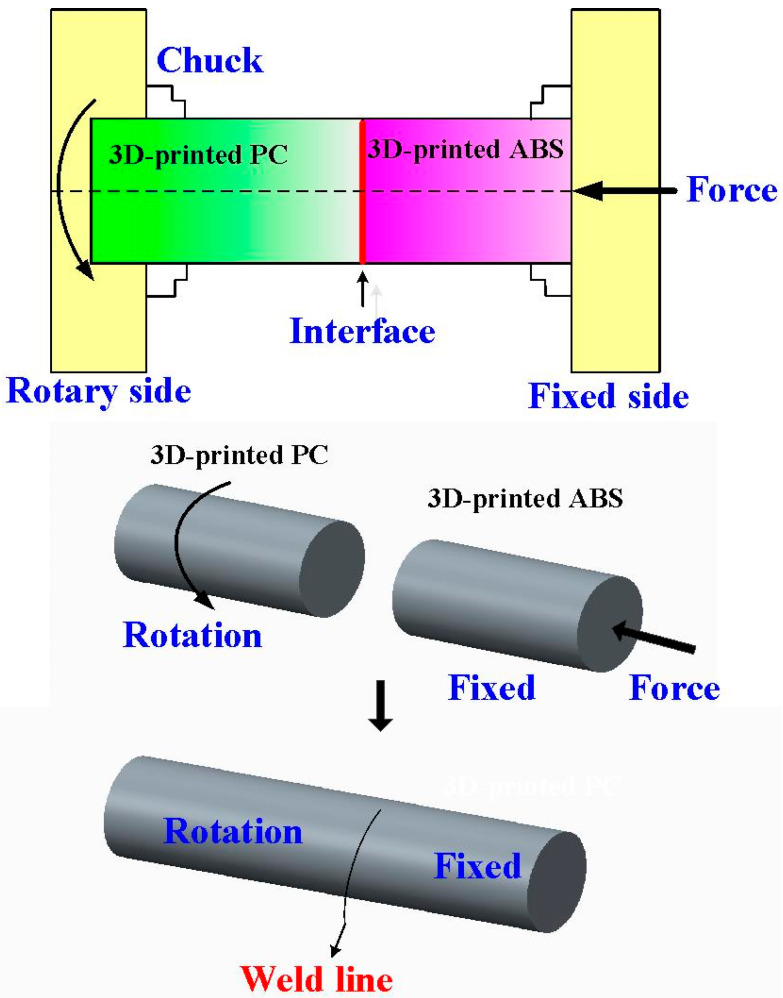
Schematic illustration of the RFW process used to manufacture both impact and bending test specimens.

Figure 1 shows the flowchart of the research process in this study. Figure 2 shows the welding specimens for RFW of ABS and PC dissimilar polymer rods. Both impact and bending test specimens are cylindrical rods with a diameter of 20 mm and a length of 40 mm. Figure 3 shows the schematic illustration of the RFW process used to manufacture both impact and bending test specimens. The welding specimens were printed with a three-dimensional printing apparatus named fused deposition modeling (FDM) (Teklink smart solution Inc., New Taipei City, Taiwan) [28] with two different kinds of thermoplastic filaments, i.e., ABS (Thunder 3D Inc., New Taipei City, Taiwan) and PC (Thunder 3D Inc., New Taipei City, Taiwan). The FDM process parameters for manufacturing PC polymer rods are bed temperature at 100 °C, printing speed of 80 mm/s, printing temperature of 245 °C, and layer thickness of 0.4 mm. The FDM process parameters for ABS polymer rods are a bed temperature of 100 °C, printing speed of 80 mm/s, printing temperature of 230 °C, and layer thickness of 0.4 mm.

**Figure 4:**
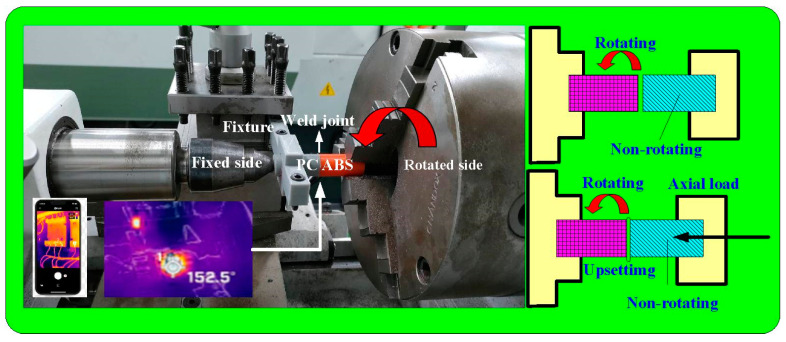
Situation of the RFW of ABS and PC dissimilar polymer rods.

**Figure 5:**
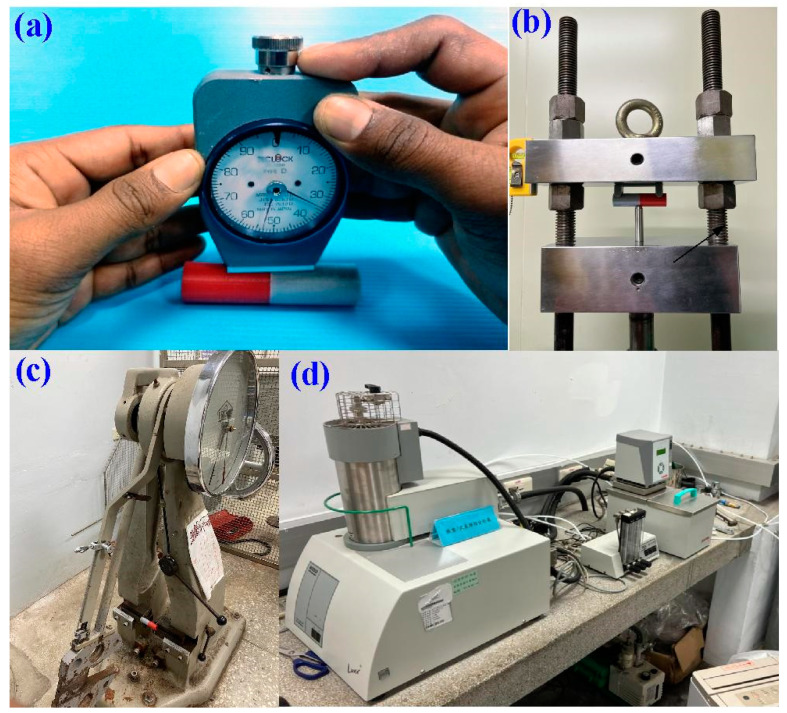
Experimental setup for (**a**) Shore A surface hardness, (**b**) bending strength, (**c**) impact energy, and (**d**) thermal analysis of the weldments.

In this work, a conventional turning machine was employed as a friction welder to perform RFW of PC and ABS dissimilar polymer rods using five rotational speeds, i.e., 330, 490, 650, 950, and 1350 rpm. The cycle time of RFW was set to 60 s, which includes a frictional time of 30 s, a welding time of 20 s, and a cooling time of 10 s. The welding parameters include axial load of 17 N, feed rate of 0.1 mm/min, and burn-off length of 2 mm. During RFW, the temperature history in the weld joint was recorded using an infrared thermal imager [29] (BI-TM-F01P, Panrico trading Inc., New Taipei City, Taiwan). After RFW, shore A surface hardness tests (MET-HG-A, SEAT Inc., New Taipei City, Taiwan), impact tests (780, Instron Inc., MA, USA), and three-point bending tests (RH-30, Shimadzu Inc., Kyoto, Japan) were carried out to evaluate the mechanical properties of the welded parts. Figure 4 shows the situation of the RFW of ABS and PC dissimilar polymer rods. Figure 5 shows the experimental setup for Shore A surface hardness, bending strength, impact energy, and thermal analysis of the weldments. To investigate the effects of ambient temperature on the mechanical properties of welded components, the specimens were placed in ten different ambient temperatures, i.e., 25, 30, 35, 40, 45, 50, 55, 60, 65, and 70 °C. The X-ray spectroscopy (D8 ADVANCE, Bruker Inc., Billerica, MA, USA) was used to characterize the phase structure of the pure ABS, pure ABS, and weld joint of ABS/PC using X-ray radiation. After bending and impact, the fracture surfaces were investigated via a stereo OM (Quick Vision 404, Mitutoyo Inc., Tokyo, Japan) and FE-SEM (JEC3000-FC, JEOL Inc., Tokyo, Japan). The heat-affected zone in the weld joint was investigated. The thermal analysis in the weld interface was characterized using differential scanning calorimetry (DSC) (STA 409 PC Luxx Simultaneous thermal analyzer, Netzsch-Geratebau GmbH Inc., Bavaria, Germany).

The temperature history in the weld joint was also investigated using COMSOL Multiphysics software. In numerical simulation software, the whole process includes setting boundary conditions, establishing the finite element mesh model, setting the parameters for RFW, setting the material nonlinear heat transfer properties, and thermal analysis of finite element. In RFW, setting boundary conditions is important for ensuring the successful joining of two materials. The boundary conditions are determined based on transient analysis. The coefficient of friction at the interface of both polymer rods was set as 0.33. The ABS polymer rod and PC polymer rod are positioned rotary-side and stationary-side, respectively. In this study, there is no temperature change at the end of rods. The temperature at the weld interface is the same for both rods. The heat generation amount between contact surfaces can be calculated according to the following equation:

Q = (K_1_fP2πN)/(L_2_ K_1_ + L_1_K_2_)

Q: heat generation amount;

K_1_: thermal conductivity of ABS (W/m-k);

K_2_: thermal conductivity of ABS (W/m-k);

f: the friction coefficient;

P: axial pressure (MPa);

N: rotational speed (rps);

L_1_: length of ABS (mm);

L_2_: length of PC (mm).

## 3. Results and Discussion

**Figure 6:**
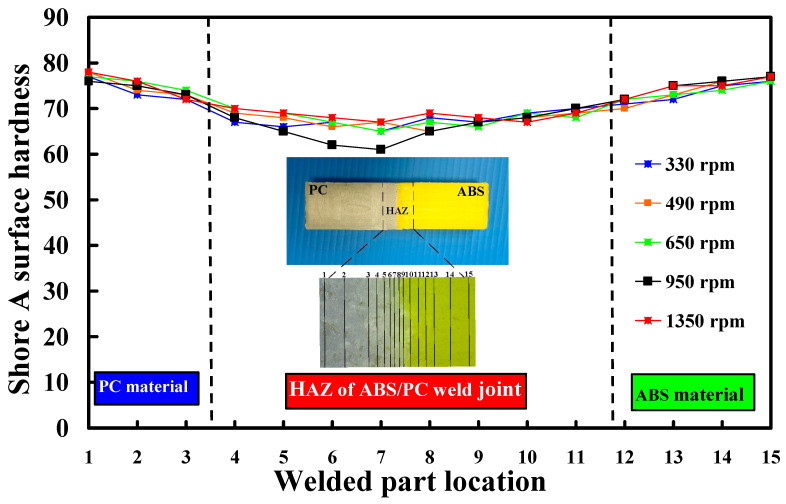
Shore A surface hardness distribution in the welded part location under five rotational speed.

**Figure 7:**
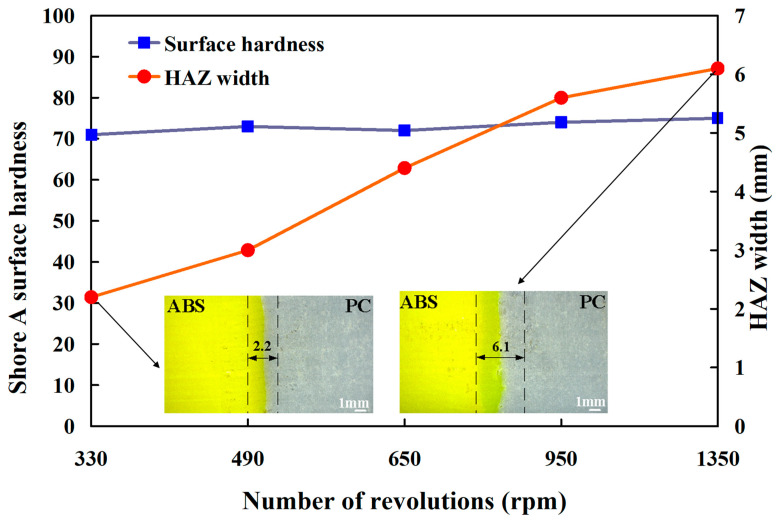
Heat-affected zone width and Shore A surface hardness under five different rotational speeds.

To investigate the Shore A surface hardness near the heat-affected zone (HAZ) of ABS/PC weld joint, the Shore A surface hardness was measured in fifteen different locations of the welded joint. Figure 6 shows the Shore A surface hardness distribution in the welded part location under five rotational speeds. This result revealed that the PC material has the highest Shore A surface hardness, followed by the ABS material. The Shore A surface hardness distribution five rotational speeds is similar. The HAZ [30] of the ABS/PC weld joint has the lowest Shore A surface hardness. The HAZ at the weld joint experiences a significant change in the material properties due to exposure to high temperatures. In this region of the HAZ, the material has a phase change from a solid to a liquid and then back to a solid. The material experiences thermal stresses during this process because of the rapid heating and cooling cycles. These stresses can form some distinct defects, such as voids [31] or micro-cracks [32]. Therefore, the Shore A surface hardness of HAZ was reduced. Figure 7 shows the HAZ width and Shore A surface hardness under five rotational speeds. Two different phenomena were found. One is that the HAZ width increases with increasing rotational speeds due to peak welding temperature [33]. This significant change can be attributed to the changes in different peak temperatures. However, the Shore A surface hardness of the ABS/PC weld joint does not change with increased rotational speeds. The Shore A surface hardness in the weld joint of RFW of ABS/PC is about 70.

**Figure 8:**
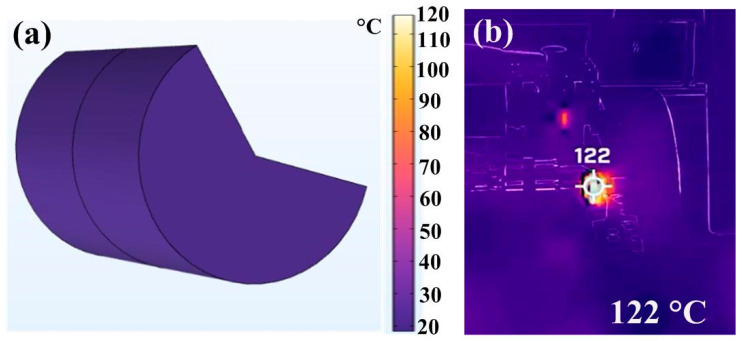
Peak temperatures in the weld interface obtained via (**a**) numerical simulation and (**b**) experiment for RFW with a rotational speed of 1350 rpm.

**Figure 9:**
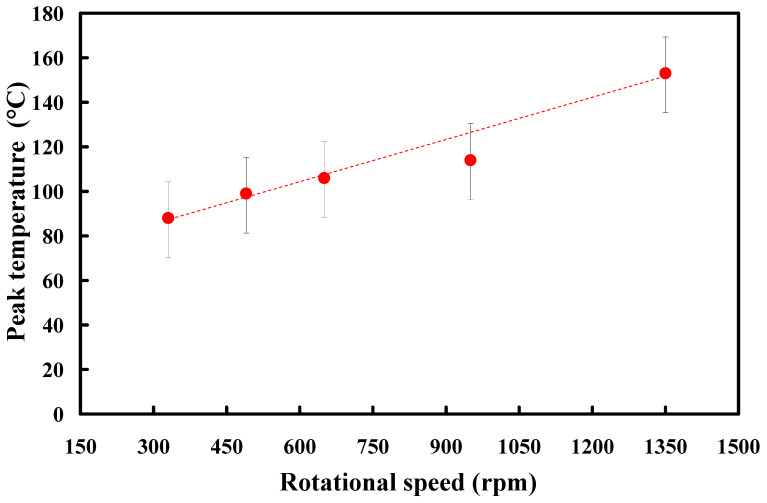
Peak temperature in the weld interface obtained via the experiment for five rotational speeds.

**Figure 10:**
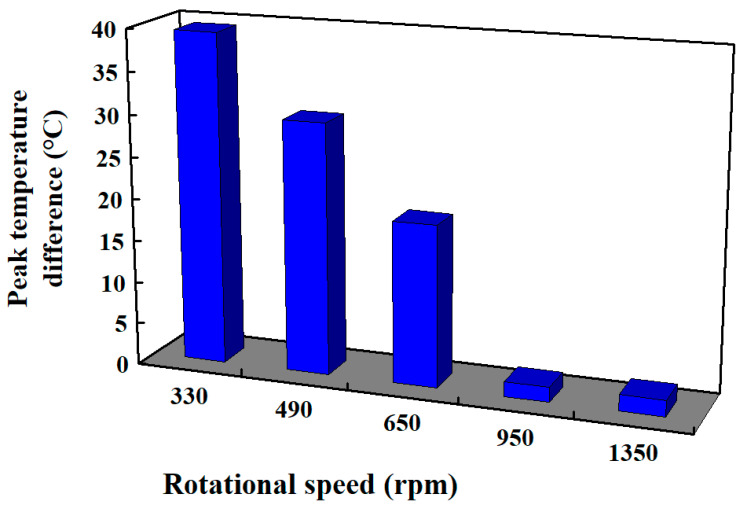
Peak temperature difference between the experiment and numerical simulation for five rotational speeds.

Figure 8 shows the peak temperatures in the weld interface obtained via numerical simulation and experiment for RFW with a rotational speed of 1350 rpm. Figure 9 shows the peak temperature in the weld interface obtained via the experiment for five rotational speeds. Significantly, the peak temperature in the weld interface (y) can be predicted by the rotational speed of (x) based on the proposed equation of y = 1 × 10^−7^x^3^ − 0.0003x^2^ + 0.2564x + 32.096 with the correlation coefficient (R^2^) of 0.9998 [34]. Figure 10 shows the peak temperature difference between the experiment and numerical simulation for five rotational speeds. The peak temperature difference between the experiment and numerical simulation for rotational speeds of 330, 490, 650, 950, and 1350 rpm is only about 39.43, 29.90, 19.25, 1.75, and 1.96 °C, respectively. It should be noted that there are some differences in peak temperature because of the difference between the experiment and simulations conditions, such as boundary conditions [35,36] and material properties.

**Figure 11:**
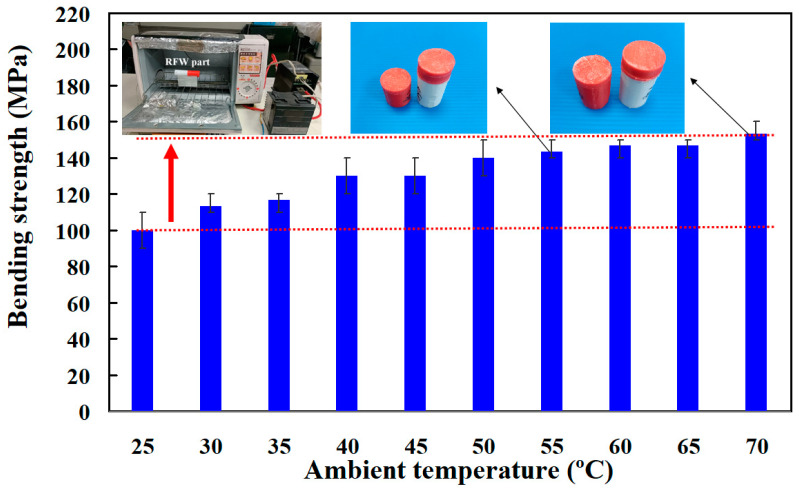
Bending strength for RFW of PC and ABS dissimilar rods under ten different ambient temperatures.

Figure 11 shows the bending strength for RFW of PC and ABS dissimilar rods under ten different ambient temperatures. In this study, two different experiments were performed. The rotational speed of the first experiment is 1350 rpm. The rotational speed of the second experiment is 950 rpm. The results showed that the ductility of the weld joint and welded part will increase when the weldments are placed at an ambient temperature above 45 °C [37]. It should be noted that the bending strength of the weldments was increased by about 53% when the weld parts were placed at 60–70 °C compared with the bending strength at room temperature. It should be noted that the bending strength obtained also has the same trend when the rotational speed is 950 rpm. The bending strength was increased by about 51% compared with the bending strength at ambient temperature. It should be pointed out that the bending strength of the welded part (y) can be predicted by the placed ambient temperature (x) based on the proposed equation of y = −0.4672x^2^ + 10.674x + 91.278 with the R^2^ of 0.9813.

**Figure 12:**
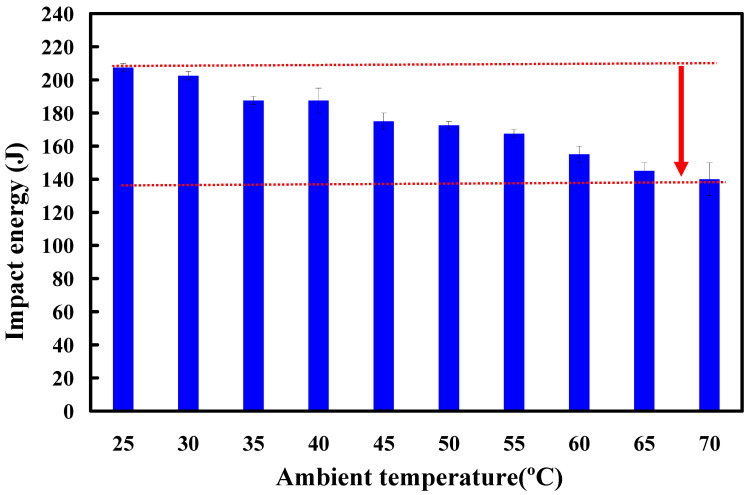
Impact energy for RFW of PC and ABS dissimilar rods under ten different ambient temperatures.

Figure 12 shows the impact energy for RFW of PC and ABS dissimilar rods under ten different ambient temperatures. The rotational speed of the first experiment is 1350 rpm. It should be pointed out that the impact energy was decreased by about 33% when the welded parts were placed at 65–70 °C compared with impact energy at room temperature. This means the softened material becomes more susceptible to deformation and failure under impact [38]. As a result, prolonged exposure to high temperatures can lead to the breakdown of the polymer chains. This degradation results in a loss of structural integrity, and a decrease in the impact strength of the weld joint. The differential expansion and contraction between the materials created stress concentrations at the joint interface when the welded part was subjected to temperature variations. These stress concentrations make it more vulnerable to failure under impact. The temperature and the impact strength are inversely proportional to each other and the decreasing rate of the impact strength is 7 to 8% for each 5 °C in this study. Interestingly, the impact energy obtained when the welded parts were placed in ten different ambient temperatures also has the same trend when the rotational speed is 950 rpm. The bending strength was decreased by about 43% compared with impact energy at ambient temperature. Significantly, the impact energy of the welded part (y) can be predicted by the placed ambient temperature (x) based on the proposed equation of y = −0.0663x^2^ − 6.7557x + 213.71. The value of the R^2^ of this prediction equation is about 0.9834.

**Figure 13:**
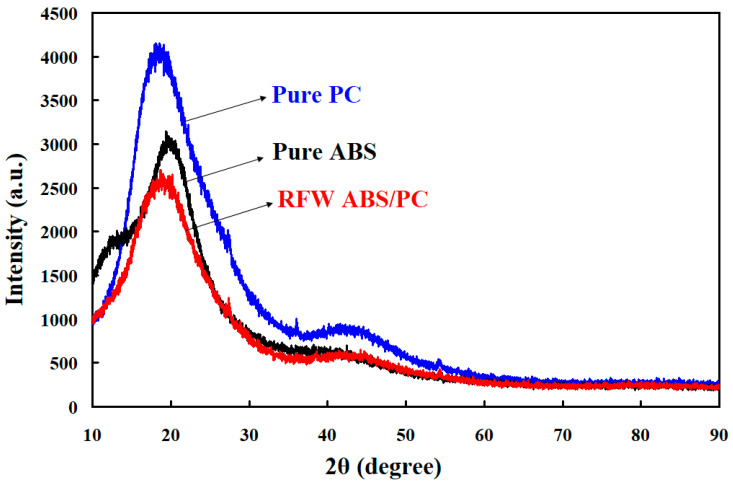
X-ray diffraction patterns of pure PC, pure ABS, and weld joint of ABS/PC.

**Figure 14:**
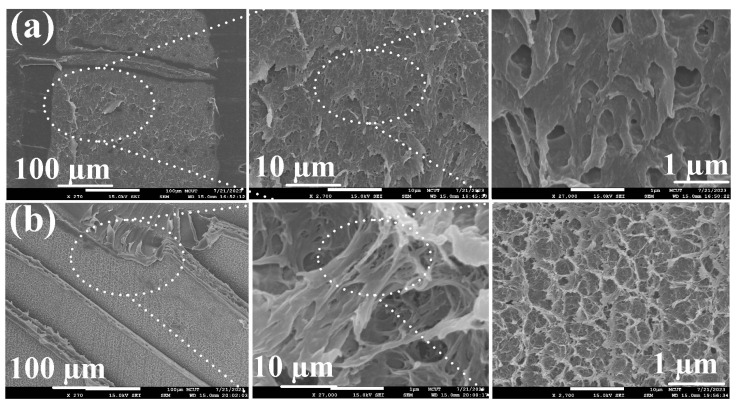
Fracture surfaces of (**a**) 3D-printed ABS polymers rod and (**b**) weld interface.

**Figure 15:**
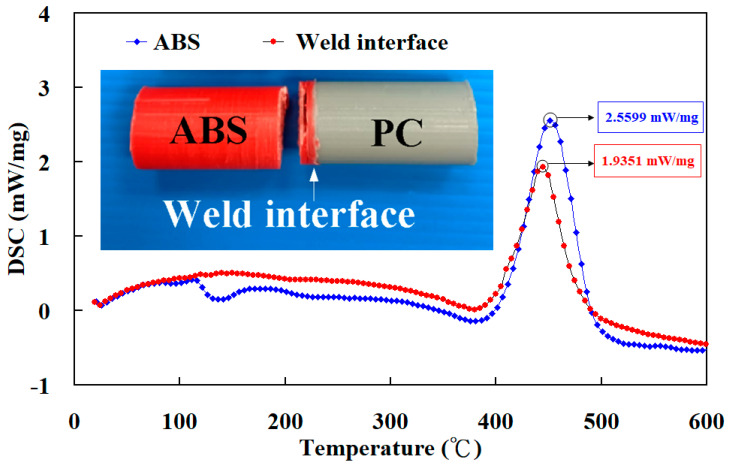
Results of the thermal analysis in the weld joint and 3D-printed ABS polymer rod.

Figure 13 shows the X-ray diffraction patterns of pure PC, pure ABS, and weld joints of ABS/PC. FDM built the pure PC and ABS. A broad feature within the range of 2θ of 12°–26° was found for the pure PC polymeric rod. A broad feature within the range of 2θ of 20° was found for the pure ABS polymeric rod. A single peak in the XRD pattern suggests that the pure PC and ABS polymeric rod is an amorphous phase [39,40], showing that the PC and ABS materials do not possess any crystalline domains. As expected, the XRD pattern also suggests that the weld joint of ABS/PC is an amorphous phase. After bending tests, a distinct result was found in that the bending fracture position of the weldment appears in the ABS side. Figure 14 shows the fracture surfaces of 3D-printed ABS polymers rod and weld interface. As can be seen, the fracture surface of the weld interface is relatively dense. The main reason is that the material of the weld interface undergoes a phase change process of high temperature melting, forging, and cooling and solidification. However, the fracture surface of the 3D-printed ABS polymers rod has some pores. These results can explain why the fractured location appears in the ABS polymer rod. This result is also confirmed by the thermal analysis using the DSC. Figure 15 shows the results of the thermal analysis in the weld joint and 3D-printed ABS polymer rod. The DSC peak appears at the temperature of 445 °C. This result shows that there is a significant thermal event occurring in the weld interface. An exothermic peak appearing between 400 and 500 °C was observed. In addition, the DSC peak appears at the temperature of 450 °C for the 3D-printed ABS polymer rod. The heat capacity for the weld interface and 3D-printed ABS polymer rod is 1.9351 mW/mg and 2.5599 mW/mg, respectively. This result shows that the weld interface has better mechanical properties than the 3D-printed ABS polymer rod due to a higher molecular orientation [41,42].

RFW is a green joining method that is ecologically friendly and energy-efficient. This study employed a conventional turning machine to perform RFW of ABS/PC. A computer numerically controlled turning machine [43] was suggested to perform the RFW of ABS/PC to reduce the human error in the RFW using a conventional turning machine because the rotational speed can be changed during RFW ABS/PC [44]. Predicting the cracks of fracture structure using the COMSOL Multiphysics software is also an important research issue [45]. This exciting topic is ongoing. This result will be presented in future work.

## 4. Conclusions

The effects of ambient temperature on the mechanical properties of frictionally welded components of ABS and PC dissimilar polymer rods were investigated experimentally and numerically. RFW was subjected to combining cylindrical or tabular components. The features of RFW involve being free from porosity or thermal distortion. In this study, 3D-printed ABS and 3D-printed PC were welded using RFW. The major conclusions from the experimental work in this study are as follows:

1. Due to the peak welding temperature, the HAZ width increases with increased rotational speeds. The Shore A surface hardness of the ABS/PC weld joint does not change with increased rotational speeds. The Shore A surface hardness in the weld joint of RFW of ABS/PC is approximately Shore A 70.
2. The bending strength of the welded part (y) can be predicted by the placed ambient temperature (x) according to the proposed equation of y = −0.4672x^2^ + 10.674x + 91.278. The bending strength was increased by about 53% when the weld part was placed at 60–70 °C compared with the bending strength at room temperature.
3. The impact energy (y) can be predicted by the placed ambient temperature (x) according to the proposed equation of y = −0.0663x^2^ − 6.7557x + 213.71. The impact energy was decreased by about 33% when the weld part was placed at 65–70 °C compared with the bending strength at room temperature.
4. The peak temperature in the weld interface (y) can be predicted by the rotational speed of (x) according to the proposed equation of y = 1 × 10^−7^x^3^ − 0.0003x^2^ + 0.2564x + 32.096.

## Data Availability

Not applicable.
